# Outcomes of Adolescent and Adult Patients with Lung Metastatic Osteosarcoma and Comparison of Synchronous and Metachronous Lung Metastatic Groups

**DOI:** 10.1371/journal.pone.0152621

**Published:** 2016-05-11

**Authors:** Ayse Gok Durnali, Fatma Paksoy Turkoz, Fisun Ardic Yukruk, Saadet Tokluoglu, Omer Kamil Yazici, Ayse Demirci, Oznur Bal, Selay Gundogdu Buyukbas, Onur Esbah, Berna Oksuzoglu, Necati Alkis

**Affiliations:** 1 Department of Medical Oncology, Dr. A.Y. Ankara Oncology Training and Research Hospital, Ankara, Turkey; 2 Department of Medical Oncology, Bahcesehir University, Istanbul, Turkey; 3 Department of Pathology, Dr. A.Y. Ankara Oncology Training and Research Hospital, Ankara, Turkey; 4 Department of Medical Oncology, Guven Hospital, Ankara, Turkey; University of North Carolina School of Medicine, UNITED STATES

## Abstract

Osteosarcomas with lung metastases are rather heterogenous group. We aimed to evaluate the clinicopathological characteristics and outcomes of osteosarcoma patients with lung metastases and to compare the synchronous and metachronous lung metastatic groups. A total of 93 adolescent and adult patients with lung metastatic osteosarcoma, from March 1995 to July 2011, in a single center, were included. Sixty-five patients (69.9%) were male. The median age was 19 years (range, 14–74). Thirty-nine patients (41.9%) had synchronous lung metastases (Group A) and 54 patients (58.1%) had metachronous lung metastases (Group B). The 5-year and 10-year post-lung metastases overall survival (PLM-OS) was 17% and 15%, respectively. In multivariate analysis for PLM-OS, time to lung metastases (p = 0.010), number of metastatic pulmonary nodules (p = 0.020), presence of pulmonary metastasectomy (p = 0.007) and presence of chemotherapy for lung metastases (p< 0.001) were found to be independent prognostic factors. The median PLM-OS of Group A and Group B was 16 months and 9 months, respectively. In Group B, the median PLM-OS of the patients who developed lung metastases within 12 months was 6 months, whereas that of the patients who developed lung metastases later was 16 months. Time to lung metastases, number and laterality of metastatic pulmonary nodules, chemotherapy for lung metastatic disease and pulmonary metastasectomy were independent prognostic factors for patients with lung metastatic osteosarcoma. The best PLM-OS was in the subgroup of patients treated both surgery and chemotherapy. The prognosis of the patients who developed lung metastases within 12 months after diagnosis was worst.

## Introduction

Cure rates of nonmetastatic high-grade osteosarcomas have increased to 60–70% by addition of adjuvant and neoadjuvant multiagent chemotherapy to surgery [1]. Despite the development of effective treatment protocols, 30–40% of cases still relapse (metachronous metastases) and more than 80% of relapses are in the lungs [2,3]. Long-term post-relapse survival rate is under 20% [1,2,3]. Approximately 20% of osteosarcoma patients are presented with metastatic disease at the time of diagnosis (synchronous metastases) and the most common metastatic site is the lungs in more than 80% of cases [4,5]. Long-term survival in primary metastatic osteosarcomas are between 10–40% [1,4]. In treatment of metastatic osteosarcoma patients, surgical removal of all metastatic foci is essential; in rerecurrences repeated thoracotomies and metastasectomies for resectable lesions are warranted [1,6]. Although the role of second-line chemotherapy for recurrent osteosarcoma is not well defined [1], some studies found positive survival effect of second-line chemotherapy [2,7]. Radiotherapy may be considered in patients without second complete remission [2]. In animal studies, CXCR4 and CXCR7 was proposed to be targets for effective metastasis-supressive treatment [8]. In previous studies, some factors such as gender [8], number of pulmonary noduls [2,3,4,9], non-necrotic metastases [9], completeness of surgical resection of all detected tumor sites [2,3,4,6], late relapse [2,3,6,10], unilateral involvement of lung metastases [2,6], histological response to preoperative chemotherapy [10] were found to influence survival in high grade osteosarcoma patients with pulmonary metastases.

In this study, we evaluated outcomes of high-grade osteosarcoma patients with lung metastases. We also compared the synchronous and metachronous metastatic group in regard to clinicopathological characteristics and treatment outcomes.

## Patients and Methods

This study is designed as retrospective analysis of adolescent and adult high-grade osteosarcoma patients with lung metastases, either synchronous or metachronous. Patient files and computer-based registries of 348 patients, histopathologically diagnosed with osteosarcoma, who received therapy according to well-established protocols and followed-up in Department of Medical Oncology; Dr. A.Y. Ankara Oncology Training and Research Hospital between June 1995 and April 2011 were retrospectively analyzed, upon the approval of hospital’s ethic board. No written informed consent was given by participants for their clinical records to be used in this study. After the approval of the ethics committee, patient records/information was anonymized and deidentified prior to analysis.

The patients under the age of 13 year-old (36 patients), with insufficient data (83 patients), low grade intramedullary or parosteal or periosteal osteosarcoma (24 patients), extraskeletal osteosarcoma (3 patients) were excluded. Of the remaining 188 patients, 93 patients with lung metastases were included into this study. The patients were divided into 2 groups according to presentation time of lung metastases: the patients with synchronous lung metastases (Group A) and the patients with metachronous lung metastases (Group B).

Clinicopathological variables and variables related to lung involvement (e.g. time to first lung metastases, presence of metastasectomy, use of chemotherapy for lung metastases, multiplicity and laterality of lung lesions and size of the largest nodule) were recorded. For analysing age as prognostic factor, arbitrarily we used cut off 18 years. Lactate dehydrogenase (LDH) and alkaline phosphatase (ALP) levels at the time of initial diagnosis was divided into 2 groups as normal and high level, according to the normal limits of biochemistry laboratory. The group of patients with high ALP was further divided into 2 groups: up to 4 times of upper limit of normal level (ULNL) and higher than 4 times. Tumor size limits for analysis were determined as 8cm, used as staging limits in the tumor node metastasis (TNM) staging system for bone sarcomas [11]. Tumor necrosis rates after preoperative chemotherapy were grouped by using the Picci’s system [12].

Overall survival (OS) was calculated as the time (months) from the date of diagnosis to either the date of death or the date of last follow-up or the date of last contact time. Post-lung metastases overall survival (PLM-OS) was calculated as the time interval (months) between the date of death or last follow-up and the date of detection of first lung metastases. Two patients who died of unrelated causes were excluded from survival analysis.

All data was entered and analyzed using Statistical Package for Social Sciences version 15.0 (SPSS, Inc., Chicago, IL, USA). Appropriate statistical analysis was carried out with a two-sided level of 0.05 and/or 95% confidence interval (CI). Differences in demographic and clinicopathologic features of the patients with synchronous or metachronous lung metastases were examined using student T-test for continuous variables, Pearson chi-square test and Fisher’s exact test for the categorical variables. OS and PLM-OS were estimated using the Kaplan-Meier method. Differences in terms of survival within groups characterized by clinical and pathologic characteristics were tested using the log-rank test. Cox proportional hazards models were also performed to assess the relative excess risk of mortality and recurrence among ostesarcoma patients with synchronous or metachronous pulmonary metastasis and to adjust for confounding factors. To select those factors with independent significant influence on recurrence and mortality, multivariate analyses were carried out in a stepwise Cox regression model. Prior to this application, univariate analyses were performed for a preliminary exploration of marked associations.

## Results

A total of 93 high-grade osteosarcoma patients with lung metastases were analysed. Sixty-five patients (69.9%) were male and 28 patients (30.1%) were female. The median age at diagnosis was 19 years (range, 14–74). Thirty-nine patients (41.9%) had lung metastases at the time of diagnosis (Group A) and 54 patients (58.1%) had metachronous lung metastases (Group B). Patient and tumor characteristics of all patients and Group A and Group B were summarized in Table 1. In Group A, 4 patients had bone metastases in addition to lung metastases at diagnosis, in Group B 3 patients had stage 3 disease and 3 patients had bone metastases at the time of diagnosis. The most common primary bone sites were femur (49 patients, 52.7%), tibia (23 patients, 24.7%) and humerus (10 patients,10.8%). The median tumor size was 12.6cm (range, 5–38). Primary tumor was operated in 76 patients (81.7%) and 6 patients underwent surgery for local recurrences. Thirty-two patients (82.0%) in Group A received chemotherapy for lung metastatic disease as first line or perioperative chemotherapy; in Group B, 33 (61.1%) received neoadjuvant and 45 patients (90%) adjuvant chemotherapy for primary tumor. After first lung metastases detected, 24 patients (44.4%) in Group B received chemotherapy. Clinicoradiological features about lung metastases and treatment modalities after detection of lung metastases were also summarized in Table 1. Of the 93 patients, 21 (24.7%) underwent pulmonary metastasectomy. Eight patients underwent repeat pulmonary metastasectomies. Eighteen patients received perioperative chemotherapy in addition to metastasectomy. Thirty-five patients (38.9%) received only chemotherapy without metastasectomy for lung metastases.

**Table 1:** Patient and tumor characteristics, with comparison of clinicopathological characteristics between patients with synchronous lung metastases (Group A) and patients with metachronous lung metastases (Group B). Abbreviations: LDH, lactate dehydrogenase; ALP, alkaline phosphatase; ULNL, upper limit of normal level; CT, Chemotherapy.

**No. of patients (%)**				
**Variable**	**All patients (*n* = 93)**	**Group A (*n* = 39)**	**Group B (*n* = 54)**	***P***
Gender				0.906
Female / Male	28 (30.1) / 65 (69.9)	12 (30.8) / 27 (69.2)	16 (29.6) / 38 (70.4)	
Age				0.564
<18 years	28 (30.1)	13 (33.3)	15 (27.8)	
≥ 18 years	65 (69.9)	26 (66.7)	39 (72.2)	
Localization				0.564
Upper extremity	13 (14.0)	7 (17.9)	6 (11.1)	
Lower extremity	74 (79.6)	29 (74.4)	45 (83.3)	
Trunk	6 (6.5)	3 (7.7)	3 (5.6)	
Histopathological subtype				0.593
Osteoblastic	44 (51.8)	19 (54.3)	25 (50.0)	
Chondroblastic	17 (20.0)	6 (17.1)	11 (22.0)	
Fibroblastic	8 (9.4)	2 (5.7)	6 (12.0)	
Telangiectatic	10 (11.8)	4 (11.4)	6 (12.0)	
Others	6 (7.1)	4 (11.4)	2 (4.0)	
Tumor size				0.199
≤ 8 cm	13 (15.9)	3 (9.4)	10 (20.0)	
> 8 cm	69 (84.1)	29 (90.6)	40 (80.0)	
LDH				0.132
Normal	33 (48.5)	11 (37.9)	22 (56.4)	
High	35 (51.5)	18 (62.1)	17 (43.6)	
ALP				0.012
Normal	35 (43.2)	9 (25.7)	26 (56.5)	
High (< 4 x ULNL)	28 (34.6)	14 (40.0)	14 (30.4)	
High (≥ 4 x ULNL)	18 (22.2)	12 (34.3)	6 (13.0)	
Surgery for primary tumor				0.386
No	17 (18.3)	13 (33.3)	4 (7.4)	
Yes	76 (81.7)	26 (66.7)	50 (92.6)	
Type of primary tumor surgery				0.155
Limb-Sparing	29 (38.2)	7 (26.9)	22 (44.0)	
Amputation	46 (60.5)	18 (69.2)	28 (56.0)	
Others	1 (1.3)	1 (3.9)		
Percentage of necrosis (primary site)				0.100
Necrosis ≥ 90% (Good)	5 (12.5)	0	5 (17.9)	
Necrosis 60–89% (Fair)	8 (20.0)	4 (33.3)	4 (14.3)	
Necrosis < 60% (Poor)	27 (67.5)	8 (66.7)	19 (67.9)	
Laterality of lung metastases				0.234
Bilateral	57 (64.0)	27 (71.1)	30 (58.8)	
Unilateral	32 (36.0)	11 (28.9)	21 (41.2)	
Number of lung metastases				0.915
≤ 3 lesions	28 (32.2)	12 (31.6)	16 (32.7)	
> 3 lesions	59 (67.8)	26 (68.4)	33 (67.3)	
Size of largest lung nodules				0.001
≤ 1 cm	21 (29.6)	15 (51.7)	6 (14.3)	
> 1 cm	50 (70.4)	14 (48.3)	36 (85.7)	
Presence of nonpulmonary metastases				0.006
None (lung only)	71 (76.3)	35 (89.7)	36 (66.7)	
Lung and local recurrence	12 (12.9)	0	12 (22.2)	
Lung and bone	10 (10.8)	4 (10.3)	6 (11.1)	
Presence of lung metastasectomy				0.943
Present	21 (24.7)	9 (24.3)	12 (25.0)	
Absent	64 (75.3)	28 (75.7)	36 (75.0)	
**No. of patients (%)**				
**Variable**	**All patients (*n* = 93)**	**Group A (*n* = 39)**	**Group B (*n* = 54)**	***P***
Presence of CT for lung metastases				<0.001
Present	53 (63.9)	32 (84.2)	21 (46.7)	
Absent	30 (36.1)	6 (15.8)	24 (53.3)	
Treatment Modalities				0.001
None	34 (37.8)	7 (17.9)	27 (52.9)	
CT only	35 (38.9)	23 (59.0)	12 (23.5)	
Surgery only	3 (3.3)	0 (0.0)	3 (5.9)	
Surgery + CT	18 (20.0)	9 (23.1)	9 (17.6)	

Median follow-up time was 115 months (1–213 months), 71 patients (76.3%) died during follow-up. Eleven patients (11.8%) were alive and 11 (11.8%) lost to follow-up. In Group B, 29 patients (53.7%) developed lung metastases within 12 months after initial diagnosis and remaining 25 patients (46.3%) developed later. Median time to detection of first lung metastases was 11.5 months (range, 3–84) in Group B. The median OS for all patients was 20 months (95% CI, 15.4–24.6), 5- and 10-year OS were 19% and 16%, respectively. The prognostic factors affecting OS on multivariate analysis were high LDH levels (hazard ratio (HR): 3.07; 95% CI, 1.39–6.80; P = 0.006); synchronous lung metastasis (HR: 2.34; 95% CI, 1.20–4.56; P = 0.010) and pathologic response to preoperative chemotherapy (HR: 0.12; 95% CI, 0.05–3.11; P = 0.044).

**Fig 1:**
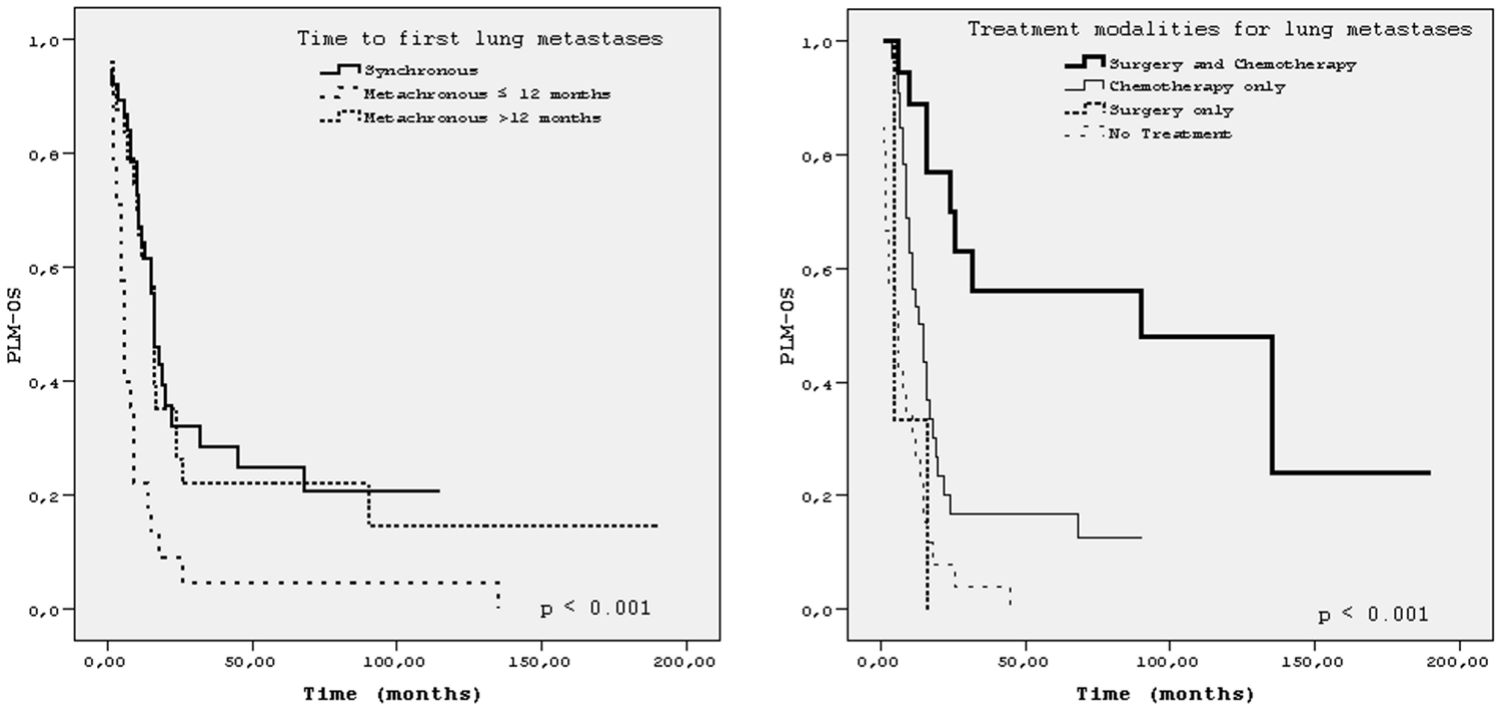
Post-lung metastases survival (PLM-OS) for the time to first lung metastses and for the treatment modalities.

**Fig 2:**
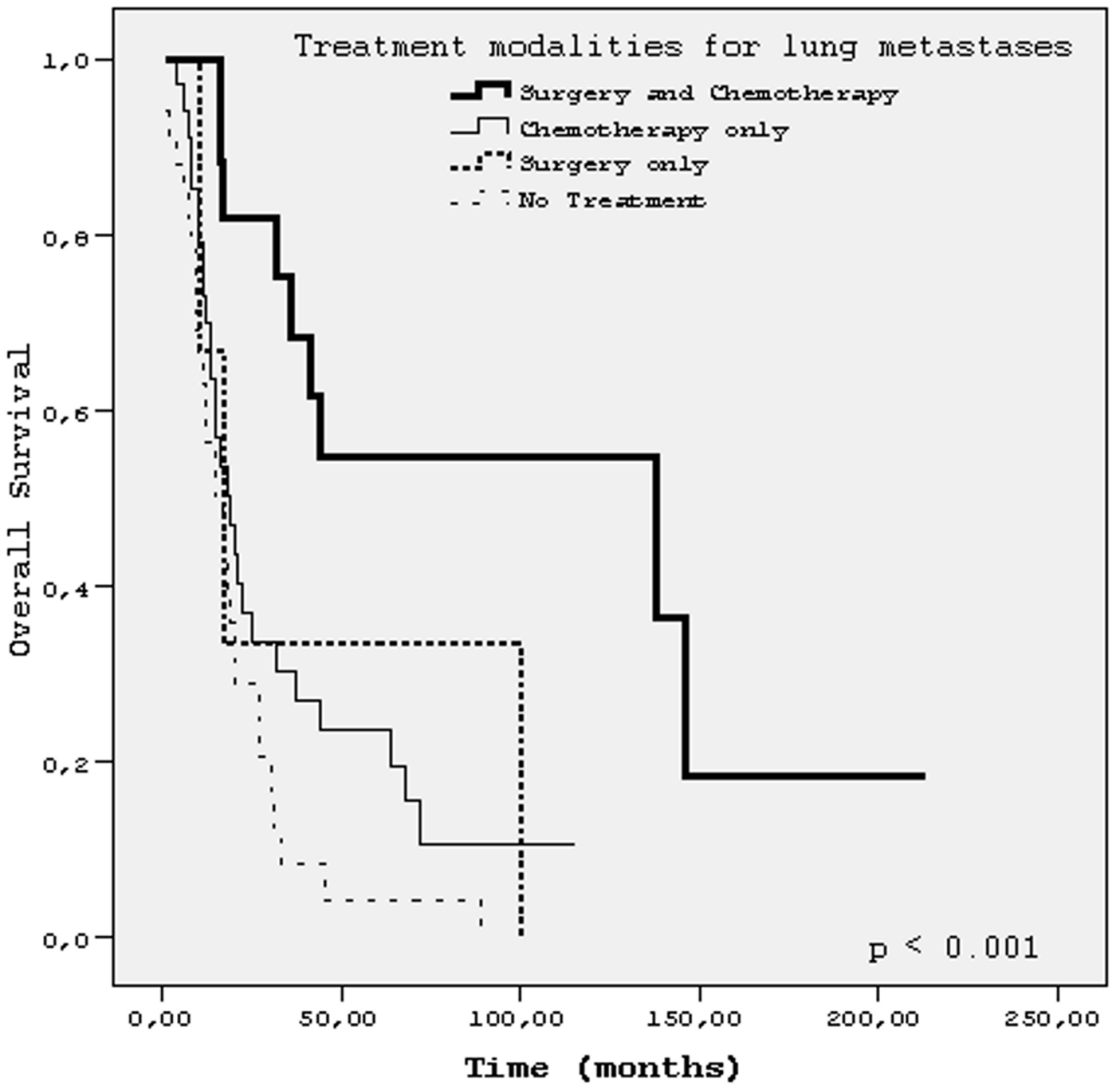
Overall survival for the treatment modalities for lung metastases.

**Fig 3:**
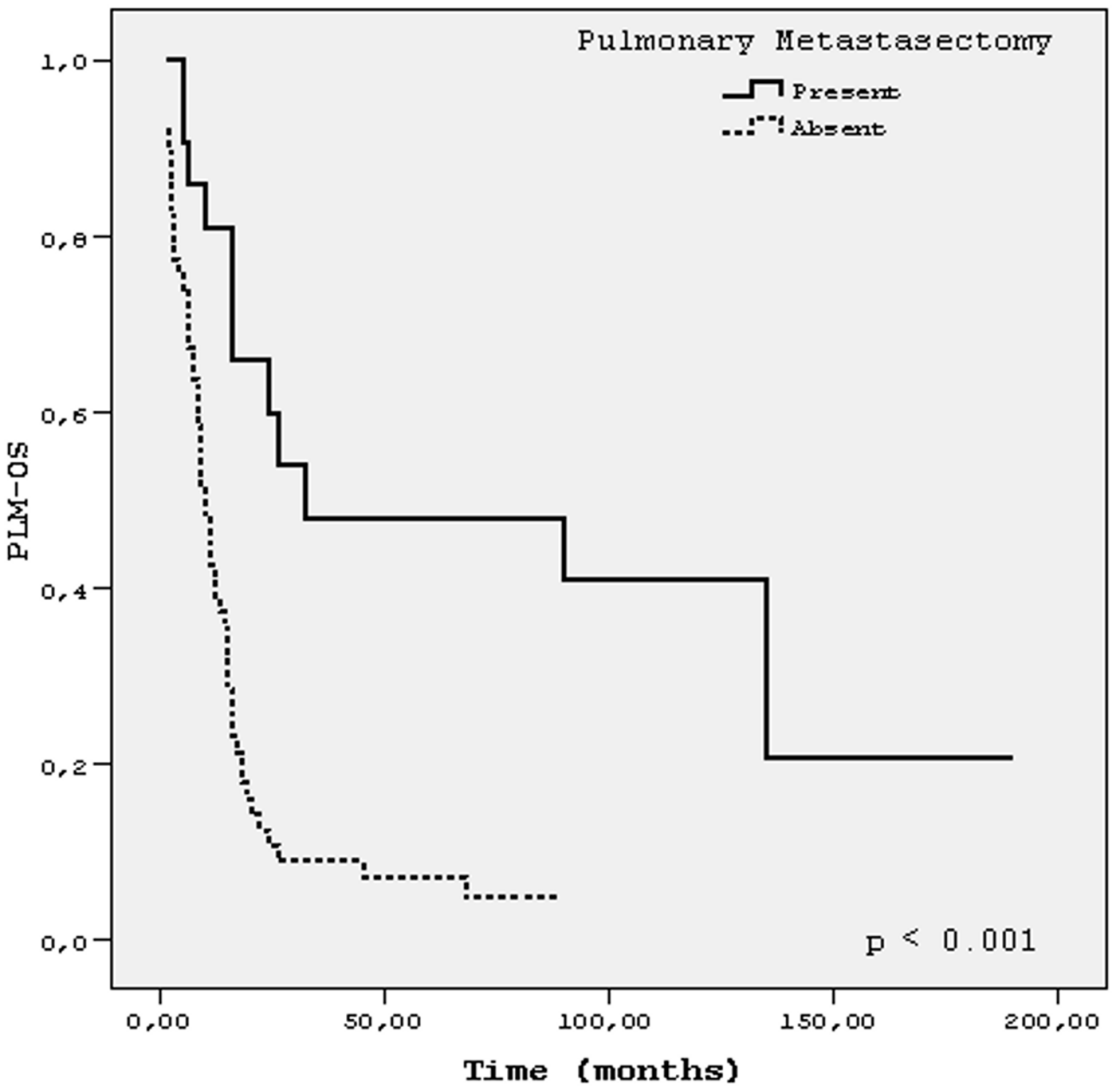
Post-lung metastases survival (PLM-OS) for the presence of pulmonary metastasectomy.

The median follow-up time after lung metastases was 91 months (1–190 months) for all patients. Five- year and 10-year PLM-OS were 17% and 15%, respectively. The median PLM-OS was 14 months (95% CI, 10.1–17.9). The best median PLM-OS (90 months) and OS (138 months) was achieved in patients treated with both surgery (metastasectomy) and chemotherapy for lung metastases, p <0.001 for both (Figs 1 and 2). Because of very small number of patients in subgroup of ‘surgery (metastasectomy) only’, we did not take this subgroup into comparison. Details of survival times of patients’ subgroups with respect to characteristics related to first lung involvement were shown in Table 2. Fig 3 showed PLM-OS curves for presence or absence of pulmonary metastasectomy. Results of univariate analysis for PLM-OS were summarized inTable 3. In multivariate analysis for PLM-OS, time to lung metastases, number of metastatic pulmonary nodules, presence of pulmonary metastasectomy, laterality of lung metastases and presence chemotherapy for lung metastases were found to be independent prognostic factors (Table 4).

**Table 2:** Overall survival and survival after first lung metastases of 93 patients. Abbreviations: OS, overall survival; PLM-OS, Post-lung metastasis overall survival; CI, coinfidence interval; CT, Chemotherapy.

Months (95% CI)				
Variable	OS, median	*P*_*log-rank*_	PLM-OS, median	*P*_*log-rank*_
Time to Lung Metastases		<0.001		<0.001
Synchronous	16 (12.0–19.9)		16 (12.0–19.9)	
Metachronous ≤ 12 months	15 (11.5–18.5)		6 (4.9–7.1)	
Metachronous >12 months	41 (28.2–53.8)		16 (14.9–17.1)	
Laterality of lung metastases		0.001		0.05
Bilateral	16 (14.2–17.8)		10 (7.2–12.8)	
Unilateral	36 (19.5–52.5)		22 (13.9–30.1)	
Number of lung metastases		0.041		0.087
≤ 3 nodules	32 (11.8–52.2)		17 (13.3–20.7)	
> 3 nodules	17 (14.3–19.7)		11 (8.2–13.8)	
Size of largest lung nodule		0.825		0.280
≤ 1 cm	22 (6.7–37.3)		18 (13.7–22.3)	
> 1 cm	19 (14.1–23.9)		10 (7.7–12.3)	
Presence of nonpulmonary metastases		0,466		0.211
None (lung only)	20 (16.5–23.5)		15 (12.3–17.7)	
Lung and local recurrence	25 (10.3–39.7)		12 (3.0–20.9)	
Lung and bone	12 (0.0–25.9)		2 (0–5.49)	
Presence of lung metastasectomy		<0.001		<0.001
Yes	100 (16.9–183.1)		32 (0.0–112.5)	
No	16 (11.9–20.1)		10 (7.6–12.4)	
Presence of CT for lung metastases		<0.001		<0.001
Yes	25(8.2–41.8)		17 (13.3–20.7)	
No	15 (9.9–20.1)		6 (3.0–8.9)	
Treatment modalities for lung metastases		<0.001		<0.001
No	15 (8.4–21.6)		6 (2.2–9.7)	
CT only	19 (12.4–25.5)		15 (9.6–20.3)	
Surgery and CT	138 (7.8–268.2)		90 (7.2–172.9)	

**Table 3:** Univariate analysis of potantial prognostic variables for overall survival after first lung metastases. Abbreviations: PLM-OS, Post-lung metastasis overall survival; HR, hazard ratio; CI, coinfidence interval; CT, chemotherapy.

Variable	PLM-OS	
	HR (95% CI)	*P*
Time to first lung metastases		< 0.001
Synchronous	1	
Metachronous ≤ 12 months	2.64 (1.45–4.91)	
Metachronous >12 months	0.91 (0.50–1.64)	
Laterality of lung metastases		0.007
Unilateral	1	
Bilateral	2.06 (1.22–3.47)	
Number of lung metastases		0.099
≤ 3 nodules	1	
> 3 nodules	1.56 (0.93–2.64)	
Size of largest lung nodule		0.294
≤ 1 cm	1	
> 1 cm	1.36 (0.77–2.40)	
Presence of pulmonary metastasectomy		<0.001
Absent	1	
Present	0.26 (0.13–0.51)	
Only CT for lung metastases		<0.001
No treatment	1	
CT, only	0.26 (0.15–0.43)	
Surgery and CT for lung metastasis		< 0,001
No treatment	1	
Surgery and CT	0.15 (0.06–0.39)	

**Table 4:** Multivariate analysis for relapse free survival and overall survival after lung metastases. Abbreviations: PLM-OS, Post-lung metastasis overall survival; HR, hazard ratio; CI, coinfidence interval; CT, Chemotherapy.

	PLM-OS	
Variable	HR (95% CI)	*P*
Time to first lung metastases		0.010
Synchronous	1	
Metachronous ≤ 12 months	1.56 (0.66–3.67)	
Metachronous >12 months	0.46 (0.19–0,97)	
Laterality of lung metastases		0.001
Unilateral	1	
Bilateral	7.4 4(2.23–24.8)	
Number of lung metastases		0.020
≤ 3 nodules	1	
> 3 nodules	4.76 (1.28–17.75)	
Presence of pulmonary metastasectomy		0.007
Absent	1	
Present	0.28 (0.12–0.71)	
Presence of CT for lung metastases		<0.001
Absent	1	
Present	0.31 (0.16–0.57)	

When clinicopathological characteristics of Group A and Group B were compared, the percentages of the patients with high LDH and high ALP levels were higher in Group A and this difference was statistically significant for ALP (p = 0.012) and the ratio of the patients who had pulmonary metastatic noduls greater than 1cm in size was higer in Group B (p = 0.003) (Table 1). In Group A, 84.2% of patients received chemotherapy for lung metastases, whereas in Group B, only 46.7% of patients received chemotherapy after lung metastases (p <0.001) (Table 1). The median OS of the patients in Group A was 16 months (95% CI, 12.0–19.9), that of the patients in Group B was 25 months (95% CI, 14.9–35.1)

The median PLM-OS of Group A was 16 months and that of Group B was 9 months. In Group B, the median PLM-OS of the patients who developed lung metastases within 12 months and who developed lung metastases later than that time were 6 months and 16 months respectively (p <0.001) (Fig 1).

## Discussion

Osteosarcomas with lung metastases are heterogenous group. In our study, the median OS for all 93 patients was 20 months; 5- and 10-year OS were 19% and 16%, respectively. In a previous study, 5-year OS for patients with lung metastatic osteosarcoma either synchronous or metachronous was 37% [13]. Tabone et al reported that OS and event-free survival were 36% and 27% at 36 months in pediatric patients who relapsed after intensive chemotherapy [14]. In other 2 studies, 5-year OS were 29% and 21% for the patients with metastases at presentation [3,4].

In this study, the median PLM-OS, for all 93 patients, was 14 months and 5- and 10-year PLM-OS were 17% and 15% respectively. Ferrari et al was reported 28% postrelapse survival at 5 years among 162 patients with initially nonmetastatic osteosarcoma [7]. In a study of Cooperative Osteosarcoma Study Group, postrelapse survival at 5 and 10 years was reported as 23% and 18% respectively [2]. In another study 10-year postrelapse survival and postrelapse event-free survival were reported as 17.0% and 11.8% respectively [6]. In the study of Aljubran et al, including patients with metachronous or synchronous lung metastatic osteosarcoma, 3-year postlung metastases survival for 85 patients was 30% [3].

In multivariate analysis for PLM-OS, time to first lung metastases, number of metastatic pulmonary nodules, presence of pulmonary metastasectomy and chemotherapy after lung metastases were found to be independent prognostic factors. In previous large studies, time to first lung metastases (usually within 1 year of diagnosis) [2,3,6,7,9,10,13,15] and pulmonary metastasectomy [2,3,6,7,9,14] were accepted as 2 main prognostic factors for pulmonary metastasized osteoarcomas. Our study is also compatible with previous reports in regard to results of multivariate analysis about laterality of lung metastases [2,6] and number of pulmonary metastatic lesions [2,7,9]. In a study among children (mean age 10.9; 28 patients with lung metastatic osteosarcoma), number, distribution and timing of lung metases, but not the size of lung metastases were found to be associated with prognosis [16].

There is no consensus about effect of chemotherapy for lung metastases. Saeter et al reported that administrating salvage chemotherapy was an independent factor for improved OS in patients with distant metastases [15]. Some studies reported that salvage chemotherapy for patients with unresectable lesions or with incomplete metastasectomy associated with a longer postrelapse or event-free survival [2,7]. Compatible with these previous reports, we found that for the patients with unresectable lesions or who refused metastasectomy, salvage chemotherapy provided better PLM-OS when compared to observation (median PLM-OS, 6 versus 15 months). In case of adding perioperative chemotherapy to metastasectomy, some studies found positive survival effect of adding chemotherapy [2,15], some did not [3,7,17]. In our study, we could not make a conclusion about this aspect due to very small number of patients who undergone only metastasectomy without perioperative chemotherapy, but with the same logic for the benefit of adjuvant/neoadjuvant chemotherapy and considering that metastatic osteosarcoma is a systemic disease, adding perioperative chemotherapy to metastasectomy seems to be rational.

In our study, clinicopathological characteristics and outcomes of Group A and Group B were compared. To our best knowledge, there is no previous study comparing clinicopathological features of the ostosarcoma patients with synchronous and metachronous lung metastases. The ratios of the patients with high baseline LDH and high baseline ALP levels were higher in Group A and this difference was statistically significant for ALP. This result might be related with tumor burden at the time of diagnosis. Percentages of the patients who had pulmonary metastatic noduls greater than 1cm in size was higher in Group B (p = 0.003). This may be explained by the fact that for staging of osteosarcoma patients chest tomography is taken at initial diagnosis, but surveillance usually includes only chest X-ray as lung imaging, or perhaps in Group B, there is more agressive subgroup of tumor. The percentages of primary tumor resections were also different between Group A and Group B (66.7% and 92.6% respectively), but this difference was not statistically significant. Chemotherapy for lung metastatic disease was given more frequently in Group A (initially diagnosed, chemo-naive patients), but frequency of pulmonary metastasectomy was similar in Group A and Group B.

The median OS of Group A was 16 months, that of Group B was 25 months. From the previous studies it has been already known that the patients with metastatic disease at diagnosis had shorter OS [18,19,20]. Therefore to compare predominantly the results about PLM-OS might be most usefull. Aljubran et al reported that there was no difference between 3-year PLM-OS of cases with synchronous versus metachronous metastases [3]. Incompatibly with this study, in our study, the median PLM-OS of Group A was higher than that of Group B significantly (median 16 and 9 months, respectively). This result might be explained with few factors: first, relapsed disease may have resistant clones resulting from prior therapy and may have more aggresivve pattern. Second, chemotherapy given for lung metastases was second line for patients in Group B, whereas it was first line chemotherapy for Group A. In other words, the patients with synchronous lung metastases are chemo-naive patients therefore they are expected to respond chemotherapy more effectively than the patients in Group B who were initially treated with neoadjuvant and/or adjuvant chemotherapy. Third, use of less intense chemotherapy for relapsed disease after detection of lung metastases in Group B may result in lower chemotherapy response and shorter survival.

In previous 2 studies, the median time to first lung metastases was reported as 10 months (range,2–56) [3] and 21 months (range, 5–60) [14]. In our study this time interval for patients in Group B was 11.5 months (range, 3–84 months). There was a marked difference between survival times of the patients in Group B who developed lung metastases within 12 months of diagnosis and after 12 months of diagnosis (for OS, median 15 months and 41 months respectively; for PLM-OS, median 6 months and 16 months respectively). The median OS of the patients who metastasized within 12 months was similar to that of the patients in Group A (15 and 16 months respectively), (Table 2). With these results, the statement that ‘osteosarcoma patients who had initially lung metastatic disease and who develop metastases within first year of diagnosis have poorer OS than the patients who develop metastases after first year’ might be more proper. In term of PLM-OS, the patients who metastasized within first year had lower PLM-OS than the patients in Group A and the patients who metastasized after first year of diagnosis. Osteosarcoma patients who metastasized within the first year of diagnosis might have more agressive pattern of disease or might have resistant clones which are able to escape from effect of first-line treatment, or can it be called as primary resistant disease? With our finding that the patients who develop metastases after first year of diagnosis and the patients in Group A had similar PLM-OS, three presumptive factors mentioned above to explain PLM-OS difference of Group A and B may not be valid for the subgroup of patients who metastasized later.

Our study has few limitations: first, this is a retropective analysis and have used previously recorded data of 16 years, so there were some missing data and also some lost to follow-up patients. Second, we had a somewhat small number of patients, this is due to rarity of adult osteosarcoma patients and excluding pediatric patients. Third, we had a heterogenous lung metastatic group including synchronous and metachronous metastases, because we planned to search and to compare clinicopathological caracteristics and outcome of all lung metastatic osteosarcoma patients.

In conclusion, PLM-OS of patients who metastasized within 12 months of diagnosis was shorter than that of the patients who metastasized after 12 months of diagnosis and who had metastases at diagnosis. The time to first lung metastases in metachrous metastatic group, the number and laterality of metastatic pulmonary nodules, the presence of chemotherapy for lung metastatic disease and pulmonary metastasectomy were found to be independent prognostic factors for PLM-OS. The best PLM-OS is in the subgroup of patients treated both surgery and chemotherapy for lung metastases. For the patients with unresectable metastases, salvage chemotherapy should be considered.
